# G4Atlas: a comprehensive transcriptome-wide G-quadruplex database

**DOI:** 10.1093/nar/gkac896

**Published:** 2022-10-16

**Authors:** Haopeng Yu, Yiman Qi, Bibo Yang, Xiaofei Yang, Yiliang Ding

**Affiliations:** Department of Cell and Developmental Biology, John Innes Centre, Norwich Research Park, Norwich NR4 7UH, UK; Department of Cell and Developmental Biology, John Innes Centre, Norwich Research Park, Norwich NR4 7UH, UK; Department of Cell and Developmental Biology, John Innes Centre, Norwich Research Park, Norwich NR4 7UH, UK; Department of Cell and Developmental Biology, John Innes Centre, Norwich Research Park, Norwich NR4 7UH, UK; National Key Laboratory of Plant Molecular Genetics, CAS Center for Excellence in Molecular Plant Sciences, Institute of Plant Physiology and Ecology, Chinese Academy of Sciences, Shanghai, China; CAS-JIC Center of Excellence for Plant and Microbial Sciences (CEPAMS), Institute of Plant Physiology and Ecology, Chinese Academy of Sciences, Shanghai, China; Department of Cell and Developmental Biology, John Innes Centre, Norwich Research Park, Norwich NR4 7UH, UK

## Abstract

RNA G-quadruplex (rG4) is a vital RNA tertiary structure motif that involves the base pairs on both Hoogsteen and Watson-Crick faces of guanines. rG4 is of great importance in the post-transcriptional regulation of gene expression. Experimental technologies have advanced to identify *in vitro* and *in vivo* rG4s across diverse transcriptomes. Building on these recent advances, here we present G4Atlas, the first transcriptome-wide G-quadruplex database, in which we have collated, classified, and visualized transcriptome rG4 experimental data, generated from rG4-seq, chemical profiling and ligand-binding methods. Our comprehensive database includes transcriptome-wide rG4s generated from 82 experimental treatments and 238 samples across ten species. In addition, we have also included RNA secondary structure prediction information across both experimentally identified and unidentified rG4s to enable users to display any potential competitive folding between rG4 and RNA secondary structures. As such, G4Atlas will enable users to explore the general functions of rG4s in diverse biological processes. In addition, G4Atlas lays the foundation for further data-driven deep learning algorithms to examine rG4 structural features.

## INTRODUCTION

RNA sequences carry not only genetic information but are also capable of folding into RNA structures to regulate sophisticated biological functions (1,2). Among all RNA structures, the RNA G-quadruplex (rG4) is one of the more important RNA structure motifs whereby a G-rich sequence can fold into four-stranded RNA G-quadruplexes via the base pairs on both Hoogsteen and Watson-Crick faces of guanines (3,4). The presence of rG4 structure motifs can be predicted from the sequence due to distinct sequence features, such as the G4 canonical structure feature of four sets of triplet guanines with three inter-loop lengths no >7 (GGGN_1–7_GGGN_1–7_GGGN_1–7_GGG) (5). In addition, there are non-canonical G3 structures such as G3 rG4s with a guanine vacancy or a bulge (labelled as G3V and G3B) and the G2 rG4s (with two G quartets) (6,7). However, rG4s that fit this sequence pattern have uncertainty in their folding state and are therefore referred to as putative rG4 (PQS). Currently, several low-throughput experimental strategies are employed to determine the folding status of PQS, such as ligand-binding assays, reverse transcriptase (RT) footprinting assays and biophysical assays (8,9). Nevertheless, due to the huge number of PQS, these low throughput approaches severely limit the discovery of rG4s across transcriptomes.

With the emergence of next-generation sequencing technologies, high-throughput transcriptome-wide rG4 detection methods first appeared in the 2016s and continue to be developed to date (9). Current transcriptome probing techniques for rG4 include rG4-seq, ligand-binding methods and chemical probing methods (7,10,11). These studies have identified a large number of rG4s across diverse transcriptomes and have also linked some to critical biological processes, such as translation and degradation (12–14).

Compared to rG4, DNA G-quadruplex (dG4) has gained more attention in recent decades, and numerous databases have emerged, such as Greglist, GRSDB, G4IPDB and G4LDB2 (15–18). Note that although dG4 and rG4 share the same sequence features, they are distinct in structural conformation, thermal stability, binding specificity and stability (19–22). Current rG4 databases include G4RNA and Plant-GQ (23,24). G4RNA has 334 *in vitro* experimentally proven individual rG4s and their corresponding predicted values (23). The volume of data in the database is severely limited because no high-throughput detection of rG4 experimental techniques is included. Plant-GQ covers 195 different plants and *in silico* predicted dG4 and rG4 but does not contain any experimentally identified rG4 data (24).

**Figure 1.**
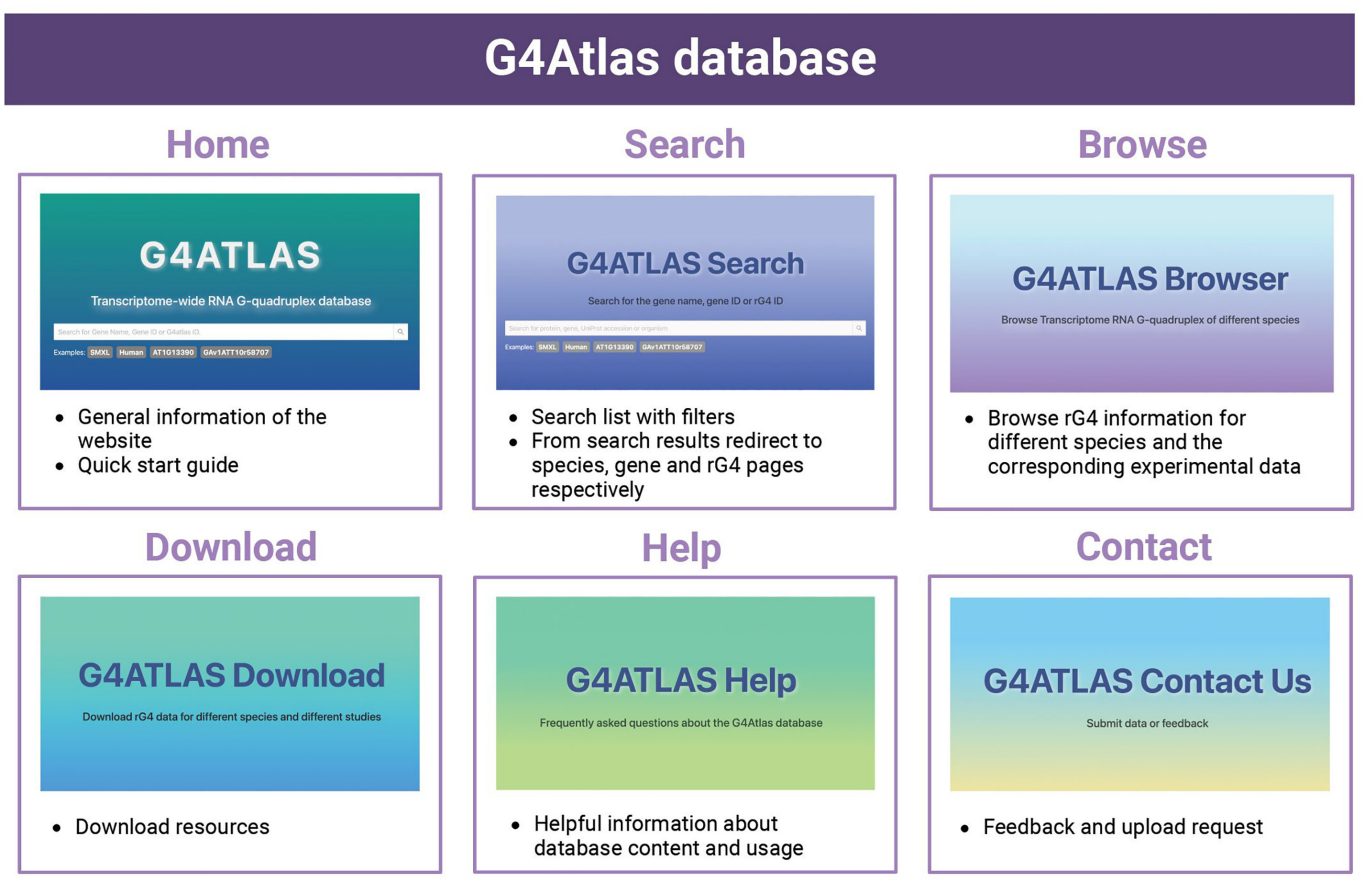
The web interface of G4Atlas database. The database consists of six main pages, including ‘Home’, ‘Search’, ‘Browse’, ‘Download’, ‘Contact’, and ‘Help’ pages. The Browse page provides access to the RG4 details page for the different species. The ‘Home’ and ‘Search’ pages provide access to rG4 information of genes and specific information for rG4s by searching for gene name, gene ID or rG4 ID. Created with Biorender.com.

Here, we present a comprehensive transcriptome-wide RNA G-quadruplex database, G4Atlas (Figure 1, https://www.g4atlas.org/). G4Atlas contains validated high-throughput experimental *in vitro* and *in vivo* rG4s for ten species, including rG4-seq, chemical probing and transcriptome ligand-binding methods (Table 1). We have incorporated data generated from methodologies across different studies, marked ‘experimentally identified rG4s’ and presented sequencing data across the rG4 regions. Furthermore, RNA secondary structure information across the rG4 regions and their flanking regions are provided to illustrate the competing folding status relationships between rG4 and RNA secondary structures. G4Atlas contains interactive charts, an all-in-one search bar, multiple data visualization approaches, extensive tips, helpful information and downloadable resources, making it an open-accessed, user-friendly transcriptome-wide rG4s database. This comprehensive database has been designed with the capacity for hosting future experimental data.

**Table 1.** Data summary of G4Atlas database

Species	Experimental methods	Treatment	References
*H. sapiens*	rG4-seq	Li^+^, K^+^, K^+^-PDS	(7)
*M. musculus* (mESC)	NAI probing	*vivo*, *vitro* (0 mM K^+^), *vitro* (150 mM K^+^)	(10)
*M. musculus* (mESC)	DMS probing	*vivo* (PDS), *vivo*, *vitro* (0 mM K^+^), *vitro* (150 mM K^+^), *vitro* (95 degree), untreat (Na^+^), untreat (Li^+^), untreat (K^+^)	
*Synechococcus* (WH8102)	DMS probing	*vitro* (0 mM K^+^), *vitro* (150 mM K^+^), untreat (Na^+^), untreat (K^+^)	
*P. putida*	DMS probing	*vivo*, *vitro* (0 mM K^+^), *vitro* (150 mM K^+^), untreat (Na^+^), untreat (K^+^)	
*E. coli*	DMS probing	*vivo*, *vitro* (0 mM K^+^), *vitro* (150 mM K^+^), untreat (Na^+^), untreat (K^+^)	
*S. cerevisiae*	DMS probing	*vivo*, *vitro* (0 mM K^+^), *vitro* (150 mM K^+^), untreat (Na^+^), untreat (K^+^)	
*H. sapiens* (Hela)	RT-stop profiling	untreat (Na^+^), untreat (K^+^)	
*H. sapiens* (HEK293T)	RT-stop profiling	untreat (Na^+^), untreat (K^+^)	
*H. sapiens*	G4RP-seq	RHPS4, BRACO, NoRX, RHPS4_BioTASQ, BRACO_BioTASQ, NoRX_BioTASQ	(11)
*H. sapiens* (Hela)	rG4-seq	Li-250 ng, K-250 ng, Li-50 ng, K-50 ng	(35)
*P. aeruginosa*	rG4-seq	Li^+^, K^+^	(41)
*E. coli*	rG4-seq	Li^+^, K^+^	
*M. musculus*	Keth-seq	Kethoxal (1 min), Kethoxal (2.5 min), Kethoxal (5 min), Kethoxal (10 min), Kethoxal-remove, No treat, Kethoxal	(37)
*M. musculus* (mESC)	Keth-seq	No treat (*vivo*), No treat (*vitro*), Kethoxal (*vivo*), Kethoxal (*vitro*)	
*H. sapiens*	Keth-seq	No treat (*vitro*), kethxoal (*in vitro*), no treat (PDS, *in vitro*), kethxoal (PDS, *in vitro*)	
*H. sapiens* (Hela)	Keth-seq	No treat, Kethoxal, No-treat.PDS, Kethoxal.PDS	
*A. thaliana*	SHALiPE-seq	*vivo*, *vitro* (K^+^), *vitro* (Li^+^)	(6)
*A. thaliana*	rG4-seq	Li^+^, K^+^, K^+^-PDS	
*O. sativa ssp. japonica*	SHALiPE-Seq	*vivo, vitro* (K^+^), *vitro* (Li^+^)	
*P. falciparum*	rG4-seq	Li^+^, K^+^, K^+^-PDS	(42)

## MATERIALS AND METHODS

### Data resource and processing

**Figure 2.**
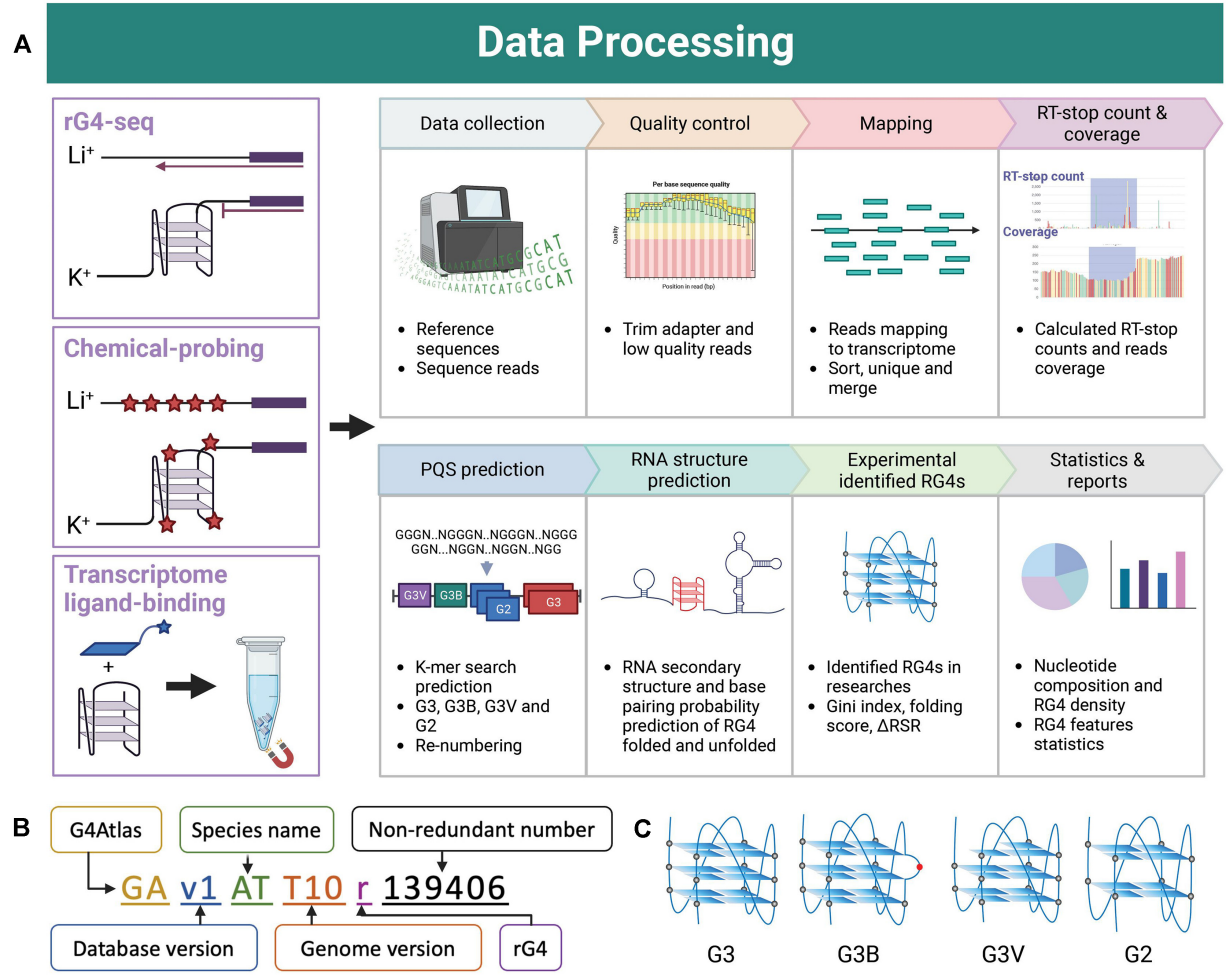
Schematic flow of data processing. (**A**) Data processing workflow. The database contains three main types of methods, rG4-seq, chemical probing and ligand-binding methods. The corresponding RG4-related information is obtained from these three data types by a standardized 8-step process on the right. In addition, each method has its own unique data processing strategy, as detailed in the Methods section. (**B**) The numbering rules for rG4 in the G4Atlas database. (**C**) Classification of rG4s. ‘G3’ is the canonical structure of rG4 of four sets of triplet guanines. ‘G3B’, ‘G3V’ and ‘G2’ are non-canonical rG4s, representing rG4s with a bulge, a guanine vacancy and four sets of 2-quartet. Created with Biorender.com.

Raw data from different research studies are collected and visualized in a database after QC, Mapping, calculation of RT-stop count, PQS prediction, RNA secondary structure prediction, rG4 for experimental identification and statistics (Figure 2A).

Species and versions covered include *Arabidopsis thaliana* (TAIR10), *Escherichia coli* (ASM584v2), *Homo sapiens* (GRCh38), *Mus musculus* (GRCm39), *Oryza sativa* (IRGSP-1.0), *Pseudomonas aeruginosa* (ASM676v1), *Plasmodium falciparum* (ASM276v2), *Pseudomonas putida* (ASM273612v1), *Saccharomyces cerevisiae* (R64-1-1) and *Synechococcus* sp.WH8102 (ASM19597v1). Genome, cDNAs and related annotation information for individual species were sourced from the Ensembl database set, including Ensembl, Ensembl Plants, Ensembl Bacteria, Ensembl Fungi and Ensembl Protists (25). The cDNA sequences were non-redundantly processed by selecting the longest isoform for each gene.

To standardize the subsequent analysis processes of rG4 experimental data, we obtained raw sequencing data from Gene Expression Omnibus (GEO) and the Short Read Archive (SRA) databases (26,27). The sequencing data were first analysed by FastQC v0.11.9 (https://www.bioinformatics.babraham.ac.uk/projects/fastqc/) for quality control and summarized by MultiQC (28). The FASTX tool (http://hannonlab.cshl.edu/fastx_toolkit/) was then applied to de-adaptors and to remove low-quality reads, depending on the experimental design of the different studies. Trimmed reads were mapped to the non-redundant transcriptome by HISAT or Bowtie as recommended by various studies, and only uniquely mapped reads were retained (29,30). Reads counts for each gene were calculated, and its state fragments per kilobase million (FPKM) values were normalized by DESeq2 (31). Genes with FPKM less than one were removed. Following the merged biological replicates of each experimental treatment, the reverse transcription stalling (RTS) signal and the reads coverage were calculated by SAMtools and Python scripts (32).

### Determination of rG4 regions

The canonical G3 rG4s sequence rule was considered to be G_3_N_1–7_, in addition to the presence of G_2_ rG4s and non-canonical G_3_ rG4s (e.g. G3 bulge) (6,7,33). We adopted rules for identifying potential rG4s, including G3, G2, G3B and G3V (6) (Figure 2C, Table 3). G4Atlas has been designed to accommodate all potential rG4 types. The 11–57 nt *k*-mer searching strategy was applied to determine each rG4 sequence region. This approach incorporates both non-greedy and greedy rG4s along with nested rG4s. We reclassified and renumbered each rG4. The numbering rules are extensible and informative, including database, species name, genome version abbreviation, rG4 label and non-redundant numbering (Figure 2B). In addition, the G4 Hunter score corresponding to each rG4 has been calculated to predict the likelihood of folding (34).

### Processing deep sequencing raw data

rG4-seq is a transcriptome-wide sequencing strategy to profile rG4 under *in vitro* conditions. The signal of reverse transcriptase stalling (RTS) in the rG4-seq database is evidential of rG4 folding (35). We adopted the data processing pipeline of the rG4-seeker (36). First, the reads start count and reads coverage of the rG4-seq data was calculated. Next, both ratios of stalled reads (RSR) and ΔRSR for each nucleotide were calculated for the *in silico* rG4 sequence regions and their upstream and downstream 50nt ranges.

$$\begin{equation*}RSR\; = \frac{{reads\;start\;count}}{{reads\;coverage}}\;\end{equation*}$$

$$\begin{equation*}\Delta RSR\; = \;RS{R_K} - \;RS{R_{Li}}\end{equation*}$$

$$\begin{equation*}\Delta RSR\; = \;RS{R_{K + PDS}} - \;RS{R_{Li}}\end{equation*}$$

Note that rG4-seq data usually contain experimental treatments for the rG4-stabilizing group (K^+^ and/or K^+^ and pyridostatin conditions) and rG4 non-stabilizing group (Li^+^ conditions), where the Li^+^ group is treated as the control, representing an unfolded rG4 status. Binomial tests of the RSR matrix were calculated to statistically assess whether the RSR difference is more significant between rG4 stabilizing conditions than under Li^+^ conditions.

Chemical probing methods are a collective name for rG4 detection by chemical reagents, including DMS profiling by dimethyl sulphate (DMS) probing and Keth-seq by N3-kethoxal probing; SHALiPE-seq and NAI-probing by 2-methylnicotinic acid imidazolide (NAI) probing. Apart from the different chemical probes adopted, these three methods are distinct. RT-stop profiling by addition of high concentrations of DMS (∼8%) allows labelling of the N7 position in the G residue (N7G) of unfolded rG4 *in vivo*. Since methylated G residues cannot be refolded in the K^+^ environment *in vitro*, *in vivo* folded rG4, i.e. regions not labelled by DMS, are foldable. Thus it is possible to infer the folding status of rG4 *in vivo* (10). Keth-seq is a high-throughput method for the detection of RNA structures, as well as rG4s (37). It determines the folding state of rG4 by identifying the presence or absence of an RT-stop signal on the G-tracts of rG4. Chemical 2-methylnicotinic acid imidazolide (NAI) can label the last G in G tracts of folded rG4 and is therefore utilized to detect the folding status of rG4s *in vitro* and *in vivo* (10,38). Both DMS and NAI are capable of penetrating the cells and thus detect the folding status of rG4 *in vivo* (6,10). Current chemical probing-related methods took advantage of the Gini index for calculating the folding score in measuring the folding status for each rG4 region.

$$\begin{equation*}{\rm{Gini\;}} = {\rm{\;}}\frac{{\mathop \sum \nolimits_{{\rm{i\;}} = {\rm{\;}}1}^{\rm{n}} \mathop \sum \nolimits_{{\rm{j\;}} = {\rm{\;}}1}^{\rm{n}} \left| {{{\rm{r}}_{\rm{i}}} - {{\rm{r}}_{\rm{j}}}} \right|}}{{2{{\rm{n}}^2}{\rm{\bar r}}}}\end{equation*}$$

$$\begin{equation*}{\rm{folding\;score\;}} = {\rm{\;}}\frac{{{\rm{Gin}}{{\rm{i}}_{{\rm{in\;vivo}}}} - {\rm{Gin}}{{\rm{i}}_{{\rm{in\;vitro\;}}\left( {{\rm{L}}{{\rm{i}}^ + }} \right)}}}}{{{\rm{Gin}}{{\rm{i}}_{{\rm{in\;vitro\;}}\left( {{{\rm{K}}^ + }} \right)}} - {\rm{Gin}}{{\rm{i}}_{{\rm{in\;vitro\;}}\left( {{\rm{L}}{{\rm{i}}^ + }} \right)}}}}\end{equation*}$$

where by *n* indicates the number of G in the rG4 and *r* denotes the RTS count in chemical probing data (6). The Gini coefficient requires high reads coverage. The default coverage threshold of the database is 50 RTS counts/nt.

### Identification of rG4

Information on the rG4 identified in the different studies was collected and collated, and labelled as ‘identified rG4’. Since most of the *r*G4s identified were non-overlapping, i.e. non-nested rG4s, we considered rG4s that overlapped with the identified rG4s and labelled them as ‘nested identified rG4s’ (Table 2). In addition to basic information on identified rG4, including gene, position and sequence, the G4Atlas database provides information on RNA secondary structure as well as its pairing probabilities, experimental raw data: including RT-stop and read coverage, and, for rG4-seq, RSR value and *P*-value for binomial tests.

**Table 2.** Statistical summary of G4Atlas database

Species	Genome version	G content	GQS density (x1000)	Potential rG4s	Samples	Treatments	Identified nested rG4s in all samples
*A. thaliana*	TAIR10	0.219	6.557	268 921	15	6	20 435
*E. coli*	ASM584v2	0.273	10.774	45 021	9	7	588
*H. sapiens*	GRCh38	0.241	13.315	1 860 403	113	25	223 426
*M. musculus*	GRCm39	0.242	11.717	1 341 657	62	22	75 509
*O. sativa*	IRGSP-1.0	0.263	14.361	2 120 209	4	3	4732
*P. aeruginosa*	ASM676v1	0.325	19.353	171 464	4	2	970
*P. falciparum*	ASM276v2	0.14	1.609	10 913	6	3	3921
*P. putida*	ASM273612v1	0.312	15.631	129 825	8	5	0
*S. cerevisiae*	R64-1-1	0.205	4.297	21 673	13	5	701
*Synechococcus* (WH8102)	ASM19597v1	0.307	18.731	68 533	4	4	0

### RNA secondary structure prediction across rG4 regions

For all putative rG4s, the RNA secondary structure information across the *r*G4 regions with their upstream and downstream 50-nt flanking sequences was predicted via *ViennaRNA* (39). We also present these RNA secondary structures along with both folded rG4s and unfolded rG4s identified from the experiments. The corresponding pairing probability for each nucleotide derived from the RNA secondary structure was generated along with the minimum free energy derived by the *efn2* function (40).

### Front-end and back-end of the database

G4Atlas (https://www.g4atlas.org/) is deployed in a separate front and back-end model (Supplementary Figure S1). The front-end application of the database is implemented with the Vue3 framework (https://vuejs.org/), and the back-end is implemented with the Python FLASK framework (https://flask.palletsprojects.com/en/2.2.0/) with PostgreSQL database (https://www.postgresql.org/). The database is user-friendly and can be accessed directly from all platforms, including mobile phones, tablets, and PC, without registration. We recommend PC access for the best browsing experience.

## DATABASE CONTENT AND USAGE

### Web interface and database summary

The web interface of the database contains six main web pages (Figure 1). The ‘Home’ and ‘Search’ page provides the search functions for species name, gene name, gene ID and rG4 ID to link with their corresponding dimension of rG4 information. The "Browse’ page enables access to the experimental knowledge of the rG4s for different species by selecting along with the statistics parameters. The data resources embedded in the database can be downloaded from the ‘Downloads’ page, and the corresponding help information is available from the ‘Help’ page. Data submissions, feedback suggestions and issues can be submitted via the ‘Contact’ page for further optimization and updates.

The current version of the G4Atlas database contains ten species, 83 different experimental treatments and 238 samples (Table 1). It includes datasets from transcriptome-wide rG4 assays, containing rG4-seq, ligand-binding methods such as G4RP-seq and chemical probing methods such as Keth-seq, DMS probing, NAI probing, and SHALiPE-seq (6,7,10,11,35,37,41,42) (Table 1). With due reference to the data processing strategies of the various originating research labs, the different experimental datasets were rearranged, processed, classified, numbered, standardized and visualized and finally collated in the database (Figure 2A).

**Figure 3.**
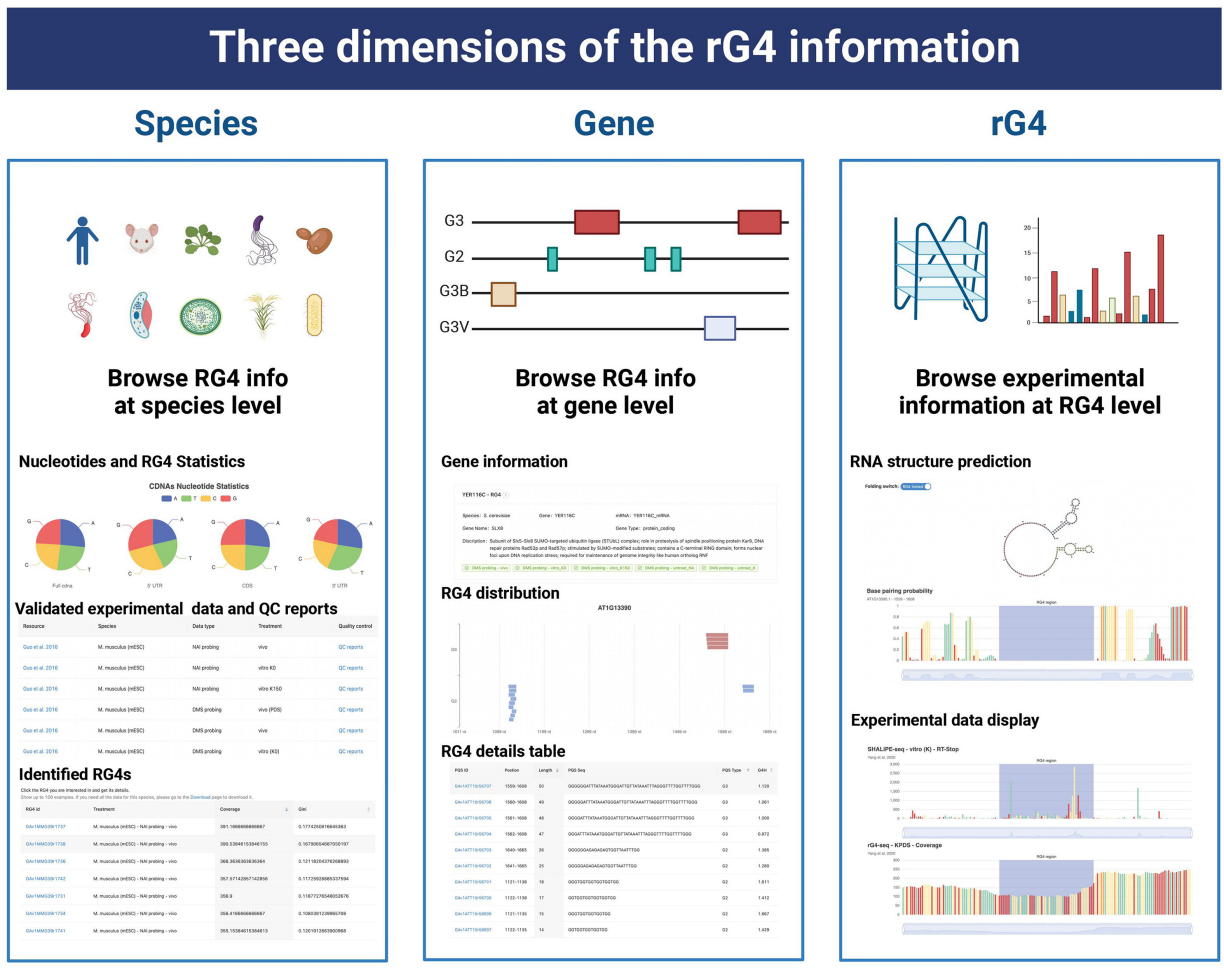
Three dimensions of rG4 information. The database provides access to rG4 information in three dimensions: species, gene and rG4. Created with Biorender.com.

The G4Atlas database allows the browsing of rG4 information in three dimensions: the species, gene and rG4 region (Figure 3). The species dimension provides access to statistical information on rG4s of the species and the transcriptome-wide experimental data for individually identified rG4s. The gene dimension presents the general information regarding the gene architecture and the locations for both putative rG4 and identified rG4s. The rG4 dimension focuses on the rG4 regions along with their flanking upstream and downstream 50nt regions, including the general information for the rG4s, *in silico* predicted RNA secondary structure across the rG4 region along with the flanking regions with and without considering folded rG4. In parallel, we included the experimental data from all the corresponding studies associated with rG4 regions.

### Browse rG4 information for diverse species

G4Atlas presents rG4 information in three dimensions: species, genes and rG4s, respectively (Figure 3). From the ‘Browse’ page, the corresponding rG4 information can be obtained by selecting the specific species. Individual species were labelled with the corresponding images and genome annotated versions along with available rG4 experimental data were included in our database. The current G4Atlas database supports ten species: with *E. coli*, *A. thaliana* and *H. sapiens* containing both rG4-seq and chemical probing data: and *H. sapiens* also containing transcriptome ligand-binding data, *M. musculus*, *O. sativa*, *P. aeruginosa*, *P. falciparum*, *P. putida*, *S. cerevisiae* and *Synechococcus* containing one of these experimental data types.

The ‘Species’ page contains four sections. ‘Species info’ provides general information about the species, including the names, the numbers of genes, the genome annotated versions and the gene types. The ‘Transcriptome statistics’ provide the ‘Nucleotides statistics’ and the ‘RNA G-quadruplex statistics’. Both nucleotides content and rG4 frequency for different genic regions (5‘UTR, CDS, 3’ UTR and full cDNA) are provided in the ‘RNA G-quadruplex statistics’ section. The ‘Experimental data’ section shows the available rG4 experimental data for the specific species in the database. The corresponding PubMed link for the related research, along with experimental material, the experimental data type and experimental treatment and the quality control report (QC report) of the experimental data, are also provided in G4Atlas.

Comprehensive, user-friendly access to rG4 experimental data is the primary function of G4Atlas. The available experimental data can be selected from the ‘Experimental data’ section. Then, the rG4s identified by the experimental data are presented in the table below. These identified rG4s were from previously published works. The rG4s that overlapped with these experimentally identified rG4s are also marked, i.e. nested rG4s. Each rG4 is linked to its rG4 dimension information, including the rG4 detailed page providing the raw experimental data for the rG4 region.

### Browse both putative and identified rG4s on genes

The locations of both putative and identified rG4s on the gene of interest will enable researchers to further explore the existence of putative rG4s and the functional importance of identified rG4s. G4Atlas provides a user-friendly all-in-one search service with input recommendations and auto-completion functions for rG4 searches (Figure 1). The user can type in a few letters of the gene name or ID number. Then the database will provide the suggested relevant search results in a dropdown list. When a search event is generated, the page will be redirected to the ‘Search page’. The ‘Search page’ displays the search results in a neat card format. The left sidebar allows the users to filter the search results by species, categories, the folding status of rG4s, and the option for selecting the target gene containing valid experimental data. Choosing the target card will lead to access to the gene dimension information.

The ‘gene details’ page contains three main sections, including gene information, experimental data, coverage, and general information regarding the rG4s on the gene (Figure 3). The ‘Gene information’ section includes available information about the gene, such as the gene name, gene number, gene type and gene description. The ‘Experimental data and coverage’ section shows the valid experimental data (normalized FPKM > 1) for the rG4s and their corresponding experimental reports, such as the related research link, the sample name, the experimental data type and the experimental treatment. The ‘rG4 statistics’ section visualizes the rG4s on the gene. rG4s in our G4Atlas database are classified into G2, G3 and G3 with a bulge (G3B) and G3 with guanine vacancy (G3V) (Figure 2C, Table 3). The different types of rG4s are marked in various colours and detailed notes and descriptions are provided. In addition, all rG4s were presented in the table with the specific rG4 index in our G4Atlas database with the rG4 position, length, sequence, type, and the G4 Hunter score. Choosing any rG4s will be redirected to the function of the rG4 dimension information.

**Table 3.** Rules for potential rG4 identification

Label	Description	Rules
G3	G3 rG4s with 1–15nt loops	G_3_N_1–15_G_3_N_1–15_G_3_N_1–15_G_3_
G2	G2 rG4s with 1–9nt loops	G_2_N_1–9_G_2_N_1–9_G_2_N_1–9_G_2_
G3B	G3 rG4s with a bulge and 1–9nt loops	G_3_N_1–9_G_2_HGN_1–9_G_3_N_1–9_G_3_ or G_3_N_1–9_G_3_N_1–9_ G_2_HGN_1–9_G_3_
G3V	G3 rG4s with guanine vacancy and 1–9nt loops	G_2_N_1–9_G_3_N_1–9_G_3_N_1–9_G_3_ or G_3_N_1–9_G_3_N_1–9_G_3_N_1–9_G_2_

Note: *N* represents the four bases, and H represents the bases other than G.

### Demonstration of specific information for rG4s

The ‘rG4 details’ page is the third dimension of our G4Atlas in presenting rG4 information. It contains more detailed information about individual rG4s, including predicted RNA secondary structure across the rG4 region and the raw experimental data. The rG4 details page has three sections. The rG4 Basic Information section includes the name of the gene in which the rG4 is located, its specific location across the gene, the rG4 type and the sequence information. Next, information on rG4 and RNA secondary structure is provided. Previous studies have proposed that RNA secondary structure might compete with rG4 folding, resulting in a potentially competitive relationship (6,7). The RNA secondary structures and the corresponding base pairing probabilities are generated across the rG4 regions with and without considering folded rG4s. A switch button can selectively swap these two states of both RNA secondary structures and the corresponding base pairing probabilities. The plots of both RNA secondary structure and base pairing probabilities are interactive and support zoom-in and out functionality, drag-and-drop chart layout conversions to enable comparisons, and download functionality. Furthermore, the four nucleotides are coloured differently, with ‘G’ highlighted in red.

The rG4 transcriptome-wide experimental raw data are provided in the form of interactive plots. Each valid experimental dataset includes at least two data metrics: RT-stop count and reads coverage. These two metrics are important for rG4 detection experiments based on reverse transcription pausing. For rG4-seq, experimental data typically contain rG4 stabilized and rG4 non-stabilized groups, such as K^+^ and K^+^ with pyridostatin (PDS) treatment groups as the stabilized groups and Li^+^ treatment groups as the destabilized groups. In the case of folded rG4, rG4-seq is typically demonstrated as a sharp drop in reads coverage downstream of the rG4 region. We combined the stabilized and destabilized groups in pairs, like K^+^ versus Li^+^ and K^+^ with PDS versus Li^+^. The folded rG4s under K + conditions are generally stronger than those identified under the K^+^ with PDS treatment. Then, the ΔRSR for each base of upstream and downstream flanking regions of rG4s and the corresponding binomial tests are presented (36). For the chemical probing methods, the reads coverage and Gini index of reverse transcriptase (RT) stop count on ‘G’ are applied as criteria for the identification of rG4s (6,10). The displayed plots are also interactive and support drag and drop sorting, zoom in and out, data type conversion, data presentation and download functionalities.

## DISCUSSION

In contrast to RNA secondary structure databases, to the best of our knowledge no RNA G-quadruplex database is currently available to the community, despite large volumes of data from transcriptome-wide high-throughput detections of rG4s emerging (7,10). The current rG4 databases, G4RNA and Plant-GQ, host only several hundred in vitro characterized rG4s or *in silico* sequence-based predictions (23,24). With recent discoveries on the functional importance of rG4s (43–48), an rG4 database with experimental validations across diverse species is in great demand. Here, we collected all the transcriptome-wide rG4 data currently available. We performed the corresponding data reprocessing, classification, normalization and visualization. Therefore, we established our comprehensive G4Atlas, an open-access, user-friendly rG4 database containing ten species, 82 experimental treatments and 238 samples, with powerful all-in-one searchable interactive graphs and a wealth of downloadable resources. Researchers can quickly and conveniently target their species and/or genes of interest and access detailed rG4 information and resources.

Nevertheless, further wet-bench and dry-bench developments are required for improving the sensitivity of rG4s detection *in vivo*. The detection of rG4s on low-abundant transcripts remains experimentally challenging due to the nature of deep sequencing technology limitations (9). The specific enrichment step could be cooperated in the experimental procedure for detecting the folding status of rG4s on low-abundant transcripts. Alternatively, both machine learning and deep learning methods (49–51) could be adopted for increasing the sensitivity of rG4 detection. The vast resources of rG4 data in our G4Atlas database is likely to facilitate the emergence of data-driven algorithms in predicting rG4 structure in the future.

## DATA AVAILABILITY

The G4Atlas database is openly accessible through https://www.g4atlas.org/ (also accessible through https://www.g4atlas.com/). Reference sequences, putative rG4, RNA secondary structure information, and identified rG4 information for all species included in the G4Atlas database are available at https://www.g4atlas.org/download.

## Supplementary Material

gkac896_Supplemental_File
