# Supplementary figures and images for: G4Atlas: a comprehensive transcriptome-wide G-quadruplex database
Source: Nucleic Acids Res. 2022 Oct 16;51(D1):D126–34. doi: 10.1093/nar/gkac896 (PMC9825586; doi:10.1093/nar/gkac896)

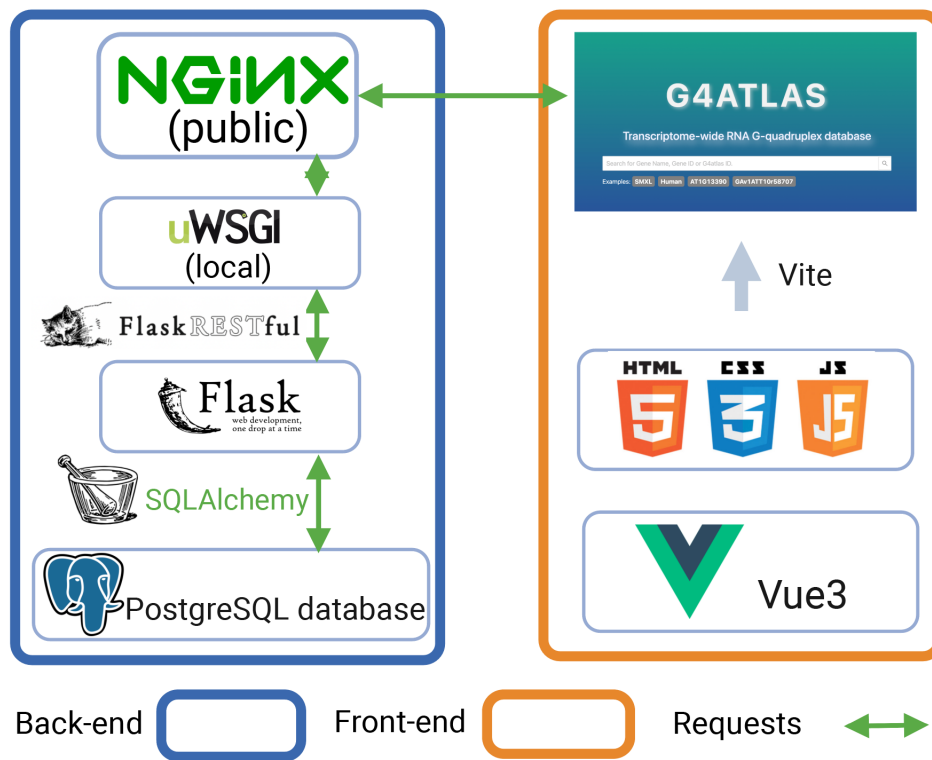

Figure S1. Front-end and back-end of the database.

Supplement: gkac896_Supplemental_File [file gkac896_supplemental_file.pdf]
